# In utero exposure to diesel exhaust is associated with alterations in neonatal cardiomyocyte transcription, DNA methylation and metabolic perturbation

**DOI:** 10.1186/s12989-019-0301-9

**Published:** 2019-04-11

**Authors:** Jamie M. Goodson, James W. MacDonald, Theo K. Bammler, Wei-Ming Chien, Michael T. Chin

**Affiliations:** 1 Division of Cardiology, Department of Medicine, University of Washington School of Medicine, Seattle, USA; 2 Department of Pathology, University of Washington School of Medicine, Seattle, USA; 3 Department of Environmental and Occupational Health Sciences, University of Washington School of Public Health, Seattle, USA; 4 Molecular Cardiology Research Institute, Tufts Medical Center, Seattle, USA; 5 Tufts Hypertrophic Cardiomyopathy Center and Research Institute, MCRI/CVC, Tufts Medical Center, 800 Washington Street, Box 80, Boston, MA 02111 USA

**Keywords:** Diesel, PM_2.5_, Transcription, DNA methylation, Metabolism

## Abstract

**Background:**

Developmental exposure to particulate matter air pollution is harmful to cardiovascular health, but the mechanisms by which this exposure mediates susceptibility to heart disease is poorly understood. We have previously shown, in a mouse model, that gestational exposure to diesel exhaust (DE) results in increased cardiac hypertrophy, fibrosis and susceptibility to heart failure in the adult offspring following transverse aortic constriction.

**Results:**

In this study, we have analyzed gene expression in neonatal cardiomyocytes after gestational exposure by RNA-sequencing and have identified 300 genes that are dysregulated, including many involved in cardiac metabolism. We subsequently determined that these cardiomyocytes exhibit reduced metabolic activity as measured by Seahorse extracellular flux analysis. We also surveyed for modifications in DNA methylation at global regulatory regions using reduced representation bisulfite sequencing and found hypomethylation of DNA in neonatal cardiomyocytes isolated from in utero DE exposed neonates.

**Conclusion:**

We have demonstrated that in utero exposure to diesel exhaust alters the neonatal cardiomyocyte transcriptional and epigenetic landscapes, as well as the metabolic capability of these cells. Understanding how exposure alters the developing heart through dysregulation of gene expression, metabolism and DNA methylation is vital for identifying therapeutic interventions for air pollution-related heart failure.

## Background

Exposure to particulate matter air pollution has been associated with increased incidence of cardiovascular disease [1–3], including arrhythmias [4], myocardial infarction [3] and heart failure [5]. Prenatal and early-life exposure to PM_2.5_ and PM_10_ is associated with deleterious effects, such as decreased placental mitochondrial DNA [6], decreased fetal growth [7], reduced birth weight [8] and increased newborn systolic blood pressure [9]. Currently, understanding the effects of in utero exposure to DE on newborn and early life health has become a public health concern, as multiple studies link maternal exposure to air pollution with increased risk of congenital heart defects [10, 11], childhood cancers such as retinoblastoma and acute lymphoblastic leukemia [12], and increased incidence of maternal gestational type 2 diabetes mellitus [13], which can in turn increase infant risk of metabolic and cardiac disorders [14]. Our lab has shown that in utero exposure to DE in mice predisposes the male offspring to heart failure after transverse aortic constriction (TAC) surgery [15].

There is growing evidence of alterations in epigenetic control due to PM_2.5_ exposure. PM_2.5_ and PM_10_ exposure have been correlated with significantly decreased global DNA methylation in blood cells as indicated by LINE1 and Alu methylation [16–18], as well as changes in targeted loci such as decreased methylation in the iNOS promoter [17] and increased methylation at the p16 promoter [19]. Diesel exhaust exposure specifically has also been shown to cause dysregulation of epigenetic signatures. Human bronchial epithelial cells exposed to diesel exhaust particulates via culture showed dysregulation of > 60% of microRNAs [20], and BALB/c mice exposed to diesel exhaust and given allergenic stimuli showed hypermethylation of the interferon-gamma promoter and hypomethylation of interleukin-4 in T-cells [21]. We have also previously shown hypomethylation in exon 1 of GM6307 in neonatal cardiomyocytes (NCMs) resulting from in utero exposure to DE, which correlates with dysregulation of the contiguous miR133a-2 gene [22].

We have previously identified transcriptional dysregulation in the hearts of male adult mice exposed in utero to DE [22]. To hone in on the early changes that might underlie the previously observed later life susceptibility to heart failure, we have performed RNA-sequencing on NCMs isolated from postnatal day 0 (PND0) offspring exposed to either filtered air (FA) or DE in utero. We observed a large number of transcriptional changes associated with in utero exposure to DE, many of which affect genes involved in normal cardiac metabolism. We have also determined the baseline metabolic phenotype of neonatal cardiomyocytes after gestational exposure and have found that DE exposure is associated with metabolic dysregulation. Additionally, we have performed reduced representation bisulfite sequencing (RRBS) on NCMs, and have found diminished methylation at CpG regions in DE-exposed NCMs. This study focuses on male mice based on our previous findings of in utero DE exposure affecting male adult cardiac outcomes [15, 23], and our observation that in utero DE exposure did not result in any change in female adult cardiac outcomes (C. Weldy, Y. Liu and M.T. Chin, unpublished data). This study is the first of its kind to show widespread cardiomyocyte changes in DNA methylation, gene transcription and metabolism as a result of in utero exposure to DE.

## Results

### In utero exposure to DE results in altered gene expression

**Fig. 1:**
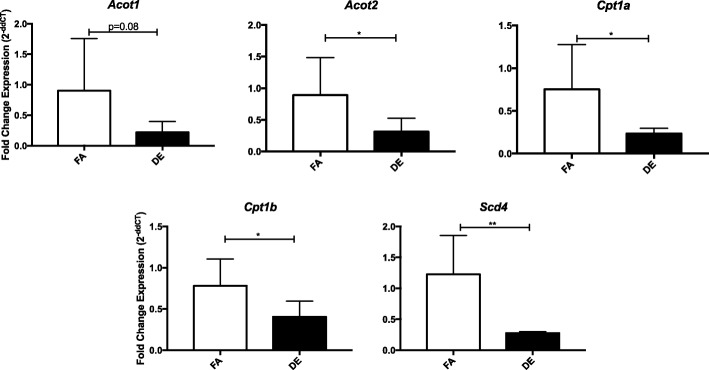
qPCR validation of select top scoring genes. qPCR was performed in the RNA samples used in sequencing plus 2 additional unsequenced samples in each group for the genes *Acot1*, *Acot2*, *Cpt1a*, *Cpt1b*, and *Scd4*. The fold change in expression is represented as 2^-deltadeltaCT (ddCT). * indicates *p*-value < 0.05, ** < 0.01

To assess alterations in transcription due to in utero exposure to DE (dams were exposed to a time weighted hourly average of 53 μg/m^3^ diesel exhaust-created PM_2.5_, 5 days a week from embryonic day 0.5 (E0.5) to E17.5), RNA-seq was performed on isolated NCMs from FA and DE PND0 offspring. No effects on birth weight, litter size or sex were observed (data not shown). We identified 300 transcripts that were significantly differentially expressed between FA and DE exposure. Table 1 shows the top ten genes with differential expression between exposure groups. Table 2 shows the top ten pathway hits using Qiagen’s Ingenuity Pathway Analysis, with arrows indicating the direction of expression change for genes identified as altered. In both, we can see a clear link between exposure and altered transcription of genes that affect cellular metabolism, showing a consistent decrease in expression in the DE NCMs. Additionally, we observe that many genes involving cell cycle control and cardiomyocyte function were affected. Gene expression differences were validated by qPCR for a select number of top scoring genes, shown in Fig. 1.

**Table 1:** Top ten specific genes showing differential expression due to in utero DE exposure

Gene Symbol	Gene Name	Log fold change	FDR
*Acot1*	acyl-CoA thioesterase 1	−2.25	2.30E-50
*Slc25a34*	solute carrier family 25, member 34	− 3.32	1.34E-39
*Acot2*	acyl-CoA thioesterase 2	−1.80	4.11E-36
*Scd4*	stearoyl-coenzyme A desaturase 4	−2.67	5.93E-34
*Tfrc*	transferrin receptor	−1.70	8.60E-21
*Hsdl2*	hydroxysteroid dehydrogenase like 2	−1.37	6.50E-19
*Hmgcs2*	3-hydroxy-3-methylglutaryl-Coenzyme A synthase 2	−4.98	1.34E-18
*Cpt1a*	carnitine palmitoyltransferase 1a, liver	−1.49	6.29E-17
*Angptl4*	angiopoietin-like 4	−2.06	7.27E-15
*Mlycd*	malonyl-CoA decarboxylase	−1.24	7.00E-12

**Table 2:** Top ten pathways showing significantly altered transcription as identified by Ingenuity Pathway Analysis (Qiagen). Arrows indicate expression in DE samples compared to FA

Ingenuity Canonical Pathway	*p*-value	Molecules
Mitochondrial L-carnitine Shuttle Pathway	2.14E-06	↓*Slc27a1*, *Acsl1*, *Cpt1A*, *Cpt2*, *Cpt1B*
Fatty Acid β-oxidation	3.47E-06	↓*Slc27a1*, *Acaa2*, *Acsl1*, *Hadha*, *Eci2*, *Hadhb*
AMPK Signaling	4.68E-06	↓*Mlycd*, *Adra1B*, *Pik3r3*, *Cpt1A*, *Acacb*, *Lipe*, *Cpt2*, *Cpt1B* ↑*Adra2a*, *Ccnd1*, *Pfkl*, *Slc2a1*
Cell Cycle Control of Chromosomal Replication	2.51E-05	↑*Cdc45*, *Mcm2*, *Mcm3*, *Mcm6*, *Mcm5*
LPS/IL-1 Mediated Inhibition of RXR Function	4.17E-05	↓*Slc27a1*, *Hmgcs2*, *Aldh9a1*, *Acsl1*, *Ppargc1b*, *Fabp3*, *Cat*, *Cpt1A*, *Cpt2*, *Cpt1B* ↑*Chst12*, *Fabp5*
GADD45 Signaling	1.00E-04	↑*Ccne1*, *Ccnd1*, *Pcna*, *Cdk1*
Hepatic Fibrosis / Hepatic Stellate Cell Activation	1.70E-04	↑*A2m*, *Igfbp4*, *Pgf*, *Col27a1*, *Col5a3*, *Igfbp3*, *Col1a1*, *Ccl21* ↓*Fgf1*, *Myh6*
Ketogenesis	2.57E-04	↓*Hmgcs2*, *Hadha*, *Hadhb*
Estrogen-mediated S-phase Entry	2.57E-04	↑*Ccne1*, *Ccnd1*, *E2f1*, *Cdk1*
Oleate Biosynthesis II (Animals)	5.89E-04	↓*Scd4* ↑*Fads1*

### In utero exposure to DE-induced changes in gene expression mimic changes observed in hypertrophic NCMs

Given our observations of increased cardiac hypertrophy in adult mice following in utero exposure to DE and TAC surgery [15, 23], we wanted to compare our observed changes in NCM gene expression to other experiments examining the changes in NCM gene expression due to other hypertrophic stressors. As shown in Table 3, using a dataset examining changes in hypertrophic NCM gene expression following transgenic overexpression of CHF1/Hey2 and serum stimulation [24] (GSE14288), we can observe significant changes in the top ten altered pathways identified in Table 2 in the GSE14288 dataset. When accounting for differential expression following the direction of majority gene change noted in Table 2, we can see that the most of pathways we identified share significant changes, including the mitochondrial L-carnitine shuttle pathway, fatty acid β-oxidation and AMPK signaling. When removing the bias of directional change, we observe that all of our top ten pathways show a significant difference in the GSE14288 dataset. This demonstrates that the gene expression changes we observe in our in utero DE NCMs are also present in NCMs that undergo hypertrophic stimuli.

**Table 3:** Comparison of significant pathways from the RNA-seq dataset using gene set tests on GSE14288, a microarray analysis of mouse neonatal cardiomyocytes overexpressing CHF1/Hey2 and treated with serum to induce hypertrophy. The # Genes column reflects the number of genes in the microarray data that were tested, and the FDR is the Benjamini-Hochberg adjusted p-value for the test that, on average, the genes in the set are differentially expressed

Ingenuity Canonical Pathway	# Genes	FDR
Mitochondrial L-carnitine Shuttle Pathway	11	5.92E-06
Fatty Acid β-oxidation	23	6.26E-15
AMPK Signaling	566	1.02E-23
Cell Cycle Control of Chromosomal Replication	48	4.64E-16
LPS/IL-1 Mediated Inhibition of RXR Function	169	2.31E-30
GADD45 Signaling	24	1.00E-08
Hepatic Fibrosis/Hepatic Stellate Cell Activation	179	3.68E-20
Ketogenesis	8	1.46E-06
Estrogen-mediated S-phase Entry	44	1.01E-19
Oleate Biosynthesis II (Animals)	8	3.56E-06

### In utero exposure to DE alters neonatal cardiomyocyte oxygen consumption

**Fig. 2:**
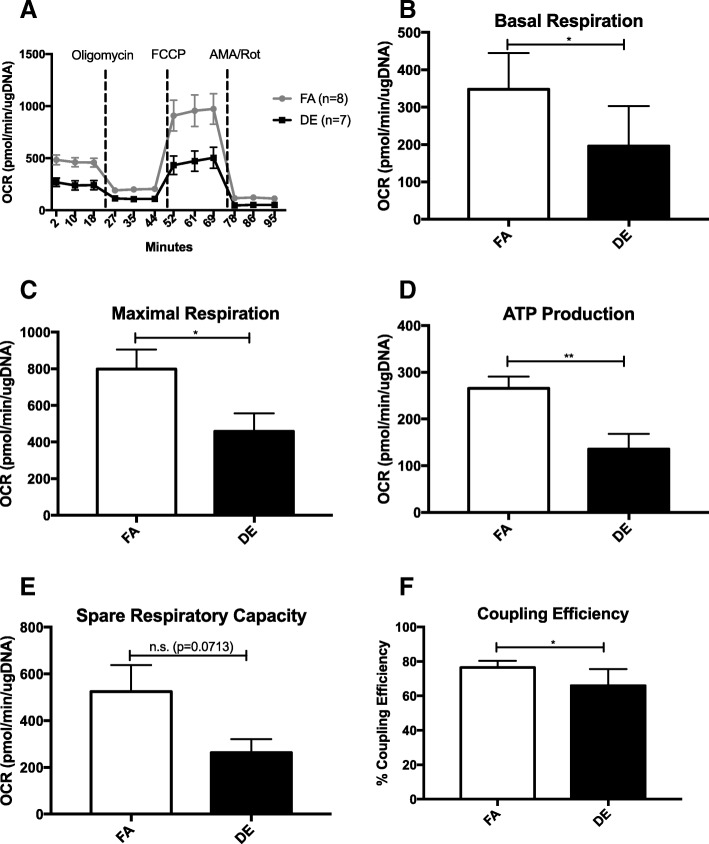
Baseline and maximal oxygen consumption rates are lower in DE NCMs. **a** shows the injection scheme and average oxygen consumption rate after injections for all FA and DE NCMs, normalized to total μg DNA. FCCP - Carbonyl cyanide-p-trifluoromethoxyphenylhydrazone, AMA/Rot – Antimycin A and Rotenone. **b**-**f** shows measurements for basal and maximal respiration, ATP production, spare respiratory capacity and coupling efficiency. * indicates p-value < 0.05, ** < 0.01

To determine whether the transcriptional changes identified in Tables 1 and 2 correlated to a functional change in the metabolic capacity of NCMs, we tested the rate of oxygen consumption of DE and FA NCMs using the Seahorse XFe24 Analyzer. However, since the switch from glucose oxidation to fatty acid oxidation in the neonatal heart doesn’t occur until sometime between postnatal day 1–7 [25, 26], we tested the response of NCMs using pyruvate/glutamate to fuel respiration. Figure 2a shows the average oxygen consumption of each group at baseline and after the injections of oligomycin, carbonyl cyanide-*p*-trifluoromethoxyphenylhydrazone (FCCP), and Antimycin A/Rotenone. After subtracting the non-mitochondrial respiration, we found that both the baseline and maximal respiration are significantly lower in the DE NCMs (Fig. 2b and c). The ATP production and coupling efficiency of the DE NCMs was also found to be significantly lower than the FA NCMs (Fig. 2d and f). DE NCMs also demonstrated a lower spare respiratory capacity, though it was not statistically significant (Fig. 2e). There was no significant difference in extracellular acidification rate between FA and DE NCMs at any of these stages (data not shown). Mitochondrial DNA content was also not significantly different between the two exposure groups (data not shown).

### In utero exposure to DE results in altered DNA methylation patterning

The temporal delay between in utero exposure, changes in adult gene expression [22] and adult onset of disease [15] suggests that an epigenetic mechanism may mediate the observed effects on adult increased susceptibility to heart failure. We performed reduced representation bisulfite sequencing (RRBS) on isolated NCMs from PND0 mice exposed to in utero FA or DE to uncover alterations in DNA methylation at CpG-rich regions, which are potential regions of genetic control. We identified 63 differentially methylated regions (DMRs), of which only 1 was hypermethylated in the DE samples. The top ten DMRs and their gene calls are represented in Table 4. To determine whether any particular pathways were being selectively altered in their DNA methylation as a result of exposure, we performed pathway analysis using Qiagen Ingenuity Pathway Analysis (Qiagen). Genes located within DMRs were given a hypothesized significant change of expression, with decreased methylation given a call of increased expression and vice versa. The top ten pathways showing altered DNA methylation are presented in Table 5. The finding of significantly reduced methylation in genes involved in pathways such as β-adrenergic, GPCR, and hypertrophic signaling suggests a mechanism by which gestational DE exposure predisposes to adult heart failure.

**Table 4:** Top ten significant DMRs in NCMs associated with in utero DE exposure

Gene	Chr.	Start	End	#CpGs	Location	AreaStat
*3110070M22Rik*	chr13	119,488,228	119,488,562	90	Intron	− 1875.26
*Grb10*	chr11	12,026,282	12,026,469	49	Intron	− 715.82
*1700030C10Rik*	chr12	20,232,946	20,234,272	36	Intergenic	− 465.28
*Mid1*	chrX	169,994,141	169,994,213	16	Intron	− 332.46
*Zfp444*	chr7	6,189,544	6,189,675	24	Exon	− 218.94
*Erdr1*	chrY	90,807,240	90,807,512	13	Intron	− 214.27
*Unc45b*	chr11	82,933,209	82,933,329	22	Intron	− 196.00
*Gm13152*	chr4	147,219,459	147,219,685	17	Intron	−138.56
*H13*	chr2	152,686,786	152,687,008	13	Intron	−118.63
*Gnas*	chr2	174,297,352	174,297,431	10	Intron	−95.56

**Table 5:** Top ten pathways with altered DNA methylation. All molecules listed have shown decreased methylation in the associated DMRs

Ingenuity Canonical Pathway	*p*-value	Molecules
Cardiac Hypertrophy Signaling	0.002	*Map3k9*, *Gnas*, *Adra2c*, *Gng12*
G_αs_ Signaling	0.002	*Rapgef2*, *Gnas*, *Gng12*
G Protein Signaling Mediated by Tubby	0.003	*Gnas*, *Gng12*
G_αi_ Signaling	0.003	*Gnas*, *Adra2c*, *Gng12*
Cardiac β-adrenergic Signaling	0.004	*Gnas*, *Gng12*, *Pde6h*
Relaxin Signaling	0.004	*Gnas*, *Gng12*, *Pde6h*
Ephrin Receptor Signaling	0.008	*Epha7*, *Gnas*, *Gng12*
CCR5 Signaling in Macrophages	0.012	*Gnas*, *Gng12*
Ephrin B Signaling	0.013	*Gnas*, *Gng12*
Protein Kinase A Signaling	0.014	*Ptpru*, *Gnas*, *Gng12*, *Pde6h*

### In utero exposure to DE significantly decreases DNA methylation in regulatory regions

**Fig. 3:**
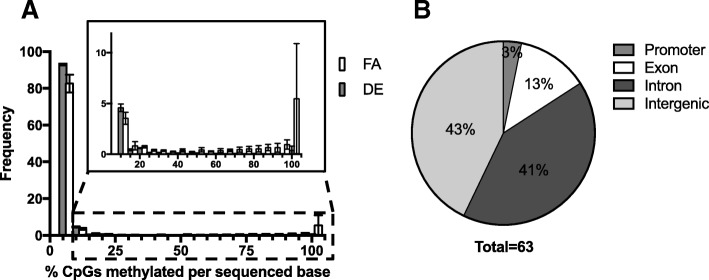
**a** Histogram of % sequences methylated within sequenced CpGs. Individual CpGs were analyzed and placed into 5% incremental bins displaying the % of CpGs from all reads methylated for a given bin. The count of CpG sites from all sequences of a given sample that show methylation in 0–5% of a samples reads are put in the 0–5% bin, and so on. The counts for each bin are averaged between samples (*n* = 4, FA and DE). Error bars are shown as standard deviation between n, which are biological replicates. **b** Genomic location of DMRs. The genomic locations of the identified DMRs were labeled as promoter (< 3000 kb upstream of TSS), intronic, exonic, or intergenic, and plotted in a pie chart showing the percentage of DMRs for a given designation

The discovery of 62 DMRs showing decreased methylation and only 1 showing an increase in the DE NCMs suggests a significant loss of methylation in the DE NCMs. This general reduction in overall DNA methylation is further demonstrated in Fig. 3a, where we examine the frequency of each CpG showing a specific percent methylation. We observe a clear decrease in the amount of methylation present in the DE samples, both through a higher proportion of 0–5% methylated CpGs and the lower amount of CpGs that were found to be methylated in > 25% of the sequences. Additionally, the genomic location of the identified DMRs is graphed in Fig. 3b, with the majority of DMRs located in intergenic and intronic regions. The genes *Gnas*, *Gng12* and *Pde6H* were heavily implicated in many of the altered pathways, and the DMRs associated with these genes were found in the intronic (*Gnas*) and intergenic (*Gng12, Pde6H*) regions.

When evaluating the individual CpGs that were identified to have significantly different methylation status between the FA and DE NCMs given a false discovery rate (FDR) of 0.1, including those that were incorporated into DMRs, we identified 12,629 differentially methylated CpGs (564 of which were included in DMRs). Of those, only 293 showed increased methylation in the DE NCMs. Assuming a null hypothesis of differential methylation affecting the samples equally, under the binomial distribution we would reject the null hypothesis with a *p*-value of 2.2e-16. Taken together, these indicate that in utero exposure to DE significantly decreases DNA methylation in CpG-rich regions.

### Comparison between transcriptional and DNA methylation alterations

In order to determine how the observed change in DNA methylation may link to the transcriptional alterations, the datasets were compared by identifying any genes within 1 Mb of a DMR that showed significant changes in expression. Surprisingly, only 14 genes were found to have significantly altered transcription near identified DMRs: *Acacb*, *Cdc45*, *Chst12*, *Cox4i2*, *Cys1*, *Etv4*, *Fkbp5*, *Glipr2*, *Gng11*, *Melk*, *Pibd1*, *Sh3gl3*, *Tubb3* and *Vwa8*. However, only *Etv4* and *Glipr2* were identified to have associated DMRs in the more stringent assessment of altered DNA methylation discussed above. Of these genes, *Acacb* and *Cox4i2* are involved in cellular metabolism, and *Gng11* is involved in GPCR signaling. Acetyl-CoA Carboxylase Beta (*Acacb*) is interesting due to its role in catalyzing the conversion of acetyl-CoA to malonyl-CoA in the fatty acid cycle, as well as being a key regulator of fatty acid oxidation through the ability of malonyl-CoA to block carnitine-palmitoyl-CoA transferase [27]. Cytochrome c oxidase subunit 4 isoform 2 (*Cox4i2*) is a subunit of the cytochrome c oxidase complex within the electron transport chain [28]. G protein subunit gamma 11 (*Gng11*) is a subunit of the GPCR signaling complex, which has also been described as a controller of cellular senescence [29]. While these genes are biologically interesting, we would expect to see a stronger link between changes in DNA methylation and changes in gene expression. It is possible that the altered DNA methylation would not manifest in transcriptional changes until the cells are stressed, or at a later age.

## Discussion

We have shown that the transcriptional profile of NCMs is significantly altered as a result of in utero exposure to DE, in large part affecting genes associated with metabolic pathways (Tables 1, 2). Metabolic dysregulation is a hallmark feature of heart failure [30], with decreased fatty acid oxidation and increased glycolysis playing an important role in late-stage heart failure [31, 32]. While it is unclear how these findings might affect adult susceptibility to heart failure, multiple studies have discovered a link between exposure to air pollution and metabolic dysregulation [33–35]. Combined with our prior findings of increased expression of *Ptprf* [22], a transcriptional change associated with decreased sensitivity to insulin signaling [36], this suggests a potential role for metabolic dysregulation as a causal link between in utero DE exposure and adult susceptibility to heart failure. Additionally, we have shown that respiratory capabilities of NCMs from in utero DE exposed neonates are hindered at both baseline and maximal respiration (Fig. 2b and c). NCMs from in utero DE exposed neonates also demonstrate decreased ATP production and coupling efficiency (Fig. 2d and f), indicative of higher energy loss and ROS production [37]. Given the known role of impaired metabolic activity on heart failure [38], these findings suggest that metabolic dysregulation during development may increase the susceptibility to heart failure at adulthood in our model. However, the persistence of the observed transcriptional alterations and the observed metabolic defects through adulthood and the causal relationships with the development of heart failure remain to be elucidated. Overall, our findings of transcriptional dysregulation in NCMs as a result of in utero DE exposure will aid in guiding the experimental focus for determining modifications in cardiac function caused by exposure.

While the observed changes in metabolic transcription and function are potentially important mediators of in utero exposure-induced susceptibility to heart failure, other mechanisms involving cardiac remodeling and fetal gene expression may be involved. One gene that showed differential expression was the alpha myosin heavy gene, *Myh6*. The RNA-seq profile showed a significant reduction in expression of *Myh6* in DE NCMs compared to FA. This gene is essential in cardiac development, with ablation experiments showing that *Myh6*^−/−^ is embryonic lethal, and *Myh6*^+/−^ mice showing impaired contractility and relaxation, as well as irregular sarcomere structure [39]. *MYH6* mutations in humans have been associated with congenital heart defects such as atrial septal defect [40], as well as in hypertrophic and dilated cardiomyopathy [41, 42]. Further exploration into the dysregulation of *Myh6* in this model would aid in understanding the potential developmental defects associated with in utero DE exposure.

Our prior findings of adult cardiovascular disease following in utero exposure suggest a temporally durable mechanism of action. Alterations of epigenetics through DNA hypomethylation have been implicated in the development and progression of cardiovascular disease [43, 44], and due to the double-stranded nature of CpG methylation these changes are incredibly stable [45]. Changes in DNA methylation both at the targeted level and genome-wide have been associated with exposure to air pollution [22, 46]. We are the first to demonstrate a change in DNA methylation at CpG-rich regions of cardiomyocytes as a result of in utero exposure to DE. The discovery of decreased methylation in DMRs associated with genes involved in GPCR and cardiac hypertrophic signaling suggests a predisposition to dysregulation in these pathways. However, there was no observed increase in transcription of *Gnas*, *Gng12*, *Adra2c* or *Pde6h* in the transcriptional profile of DE-exposed NCMs, though *Gng11* was identified to have increased expression. It is possible that the changes in transcription affected by DNA methylation would be stimulated through stress, or will manifest themselves sometime beyond the neonatal period. GPCR signaling also plays a role in the autonomic nervous system control of cardiac regulation. Considering our prior findings of decreased blood pressure at baseline in in utero DE exposed 10 week old mice [15], it is important to further understand how these molecular changes are affecting physiological outcomes in both the neonatal and adult hearts. Future studies targeting autonomic features such as heart rate variability would likely be informative.

It is unclear whether this change in DNA methylation is occurring through a directed or undirected pathway. Exposure to PM_2.5_ has been shown to have an associated increase in oxidative stress [47–49]. Passive removal of DNA methylation can occur due to oxidative damage from ROS by damaging the DNA to the extent that DNA methlyltransferases (DNMTs) are unable to bind to the lesion area, thereby averting the maintenance of methylation in daughter cells [50, 51]. If this process is occurring early in development during the repatterning of methylation, these changes would be extremely heritable, and likely occurring in multiple cell and organ types [45]. It is also possible that cellular damage due to oxidative stress prevents DNA methylation through sequestration of glutathione [52]. Targeted changes in DNA methylation in response to air pollution have been described previously. Hypermethylation of the p16 promoter in alveolar epithelial cells of mice exposed to PM_2.5_ in conjunction with increased DNMT1 expression has been reported [19] and decreased methylation on the first exon of *Gm6307*, which drives *miR133a-2* expression, has also been reported in association with in utero DE exposure [22]. Our studies of transcriptional dysregulation in NCMs did not identify any DNA methyltransferase or TET family enzyme genes as being differentially expressed, though this does not preclude the possibility of altered expression earlier in development leading to decreased methylation that was maintained until birth.

For future studies, refining the contributions of in utero DE exposure and later life cardiac outcomes will be important for focusing future experiments. Meta-analysis of epidemiological studies have demonstrated the increased risk of congenital heart defects associated with maternal exposure to nitrogen dioxide, sulfur dioxide, and particulate matter [53, 54]. Although we favor particulates as being likely responsible for the observed effects, contributions from other components cannot be ruled out. Additionally, it is not clear whether maternal effects, such as placental insufficiency brought on from inflammatory response to exposure, are the main influencers on the offspring cardiac outcomes, or if particulate and/or gaseous matter is able to cross the placental barrier and directly interact with the cardiovascular system of the developing embryos.

## Conclusions

Our study is the first to systematically analyze and demonstrate the profound effects of in utero DE exposure on cardiomyocyte gene transcription, DNA methylation and metabolism. It is unclear whether these effects are due to a direct effect of DE exposure or an indirect mechanism via the maternal response to exposure, although our previous observation that maternal exposure leads to placental inflammation [15] is consistent with an indirect mechanism. A limitation of our study is that the correlation between mRNA expression, DNA methylation and metabolic activity is imperfect. Nevertheless, these findings provide important datasets for mining the potential pathways that mediate the deleterious effects of diesel exhaust on the developing heart that predispose to heart disease. Identifying pathological pathways that promote the development of air pollution-related heart disease is a first step in developing potential therapies.

## Methods

### Diesel exhaust exposure and mice

Housing and exposure facility conditions were carried out as previously described [15, 22, 23]. C57Bl/6 J mice were used in these experiments, housed in specific pathogen free (SPF) conditions, on a 12/12-light/dark cycle. Female and male mice between the ages of 12 to 16 weeks were transferred to our Northlake Diesel Exposure Facility located near the University of Washington (UW) and housed under SPF conditions in Allentown caging systems. Female mice were paired with male mice for timed mating in FA. After observation of a vaginal plug, pregnant mice were put into FA or DE with exposures beginning at E0.5 and lasting until E17.5, at which point pregnant mice were transferred from the Northlake facility to the UW Medicine South Lake Union SPF vivarium for neonatal sample collection.

Diesel exhaust was generated from a single cylinder Yanmar diesel engine (Model YDG5500EV-6EI) operating on 82% load. A detailed analysis of DE particulate components in this system has been previously reported [55]. DE exposures were conducted for 6 h per day (9 am − 3 pm) 5 days a week (Monday – Friday) and DE concentrations were regulated to 300 μg/m^3^ of PM_2.5_. A 300 μg/m^3^ concentration of PM_2.5_ 6 hours a day, 5 days a week equates to a time weighted hourly average of 53 μg/m^3^. The exposure characteristics detailing gas, particle-bound polycyclic aromatic hydrocarbons (PAH), and particle diameter of this system have been previously reported [55, 56]. In previous measurements, oxides of nitrogen concentrations were 1800 ppb NO_x_ and 60 ppb NO_2_, carbon monoxide was 2 ppm, and carbon dioxide was 1000 ppm. The mass fraction of particle-bound PAH was 20 ng/mg PM_2.5_ and the ratio of the organic carbon to elemental carbon mass concentration was 0.10. The mass median aerodynamic diameter of particles was 85 nm (GSD 1.2) and the count median thermodynamic equivalent diameter was 87 nm (GSD 3.0). The exposure approximates real world ambient exposure in an urban setting.

### Isolation of NCMs

Upon birth, PND0 hearts were harvested, trimmed of surrounding vascular and atrial tissue, and dissociated as previously described [57, 58]. Males were detected through post-mortem dissection and identification of testes. Dissociation was performed using 1 mg/mL Liberase TH (Roche; Pleasanton, CA, USA) in 1X HBSS by incubating hearts at 37 degrees for 5 min in solution, with pipetting to release cells after incubation. Media containing released NCMs was adjusted to 20% FBS-DMEM, and cellular dissociation was continued until the majority of cells were released. Cells were then filtered using a 70 μm sieve, re-eluted in 20% FBS-DMEM with 20 μm Ara-C and incubated at 37 degrees for 1 h to allow fibroblasts to attach onto the plate. After incubation, media with suspended cardiomyocytes was carefully removed, spun and purified cardiomyocyte pellets were collected and frozen at − 80 °C.

### RNA sequencing

RNA was purified from frozen PND0 isolated NCMs using Trizol (Thermo Fisher Scientific, Waltham, MA, USA), following the manufacturer’s protocol. For each sequencing sample, NCMs from one male neonatal heart were isolated and used for each n (*n* = 3 for FA, DE). Ribosomal RNA was depleted by poly-A enrichment, and sample libraries were created using the TruSeq Stranded mRNA kit (Illumina, San Diego, CA). Each library was barcoded using the Illumina adapters, and amplified with 13 cycles of PCR. Library concentrations were quantified using the Quant-it dsDNA Assay (Life Technologies, Carlsbad, CA). Libraries were normalized and pooled based on Agilent 2100 Bioanalyzer results (Agilent Technologies, Santa Clara, CA), and the pools were sequenced on an Illumina HiSeq 2500.

To process sequences, Illumina RTA-generated BCL files were converted to FASTQ files and sequence read and base quality were checked using the FASTX-toolkit (http://hannonlab.cshl.edu/fastx_toolkit/) and FastQC (http://www.bioinformatics.babraham.ac.uk/projects/fastqc/). Sequences were aligned to mm10 with reference transcriptome Ensembl v67 using Tophat [59]. Lane level bam data files were merged using the Picard MergeSamFiles tool and suspected PCR duplicates were marked, not removed, in the alignment files using the Picard MarkDuplicates tool (http://broadinstitute.github.io/picard/). Local realignment was performed around indels, and base quality score recalibration was run using GATK tools [60]. Variant detection was performed with the GATK Unified Genotyper version 2.6.5 [61], and aligned data were used for isoform assembly and quantitation with Cufflinks [59, 62]. Fragment counts were generated using the featureCounts function in the Bioconductor Rsubread package [63]. Comparisons were performed using the Bioconductor edgeR package. Genes were identified as significantly different between groups if they had a 25% or greater difference in expression between the two groups at an FDR adjusted *p*-value of 0.05 or less. To assess consistency between our data and previous results (GSE14288) we performed self-contained gene set tests using the eleven pathways we identified using IPA (Table 2), testing for evidence that those gene sets were differentially expressed in the previous results as well. We used the limma fry function to perform the gene set tests [64].

Select genes were validated by qPCR using iTaq Universal Sybr Green Supermix (BioRad). Primers used for validation were as follows:

Acot1-For: 5′-TACGATGACCTCCCCAAGAA-3′, Acot1-Rev: 5′-AGCCCAATTCCAGGTCCTTT-3′, Acot2-For: 5′-ACAACTACGACGACCTCCCC-3′, Acot2-Rev: 5′-AGCCCAATTCCAGGTCCTT-3′, Cpt1a-For: 5′-TCCATGCATACCAAAGTGGAC-3′, Cpt1a-Rev: 5′-CGATGTTCTTCGTCTGGCTT-3′, Cpt1b-For: 5′-AGCCCCCTCATGGTGAAC-3′, Cpt1b-Rev: 5′-AGTTTGCGGCGATACATGA-3′, Scd4-For: 5′-TGGAGCCACCGAACCTATAA-3′, Scd4-Red: 5′-GGCCCATTCATACACGTCAT-3′, 18S-For: 5′-GGACAGGATTGACAGATTGATAG-3′, 18S-Rev: 5′-ATCGCTCCACCAACTAAGAA-3′. Expression was normalized to ribosomal RNA 18S. Samples were run on the 7900HT Fast Real-Time PCR System (ABI). Statistical analysis was carried out using a t-test for comparison of ddCT values.

### Metabolic capacity analysis

Cellular respiration was performed on NCMs using the mitochondrial stress test (Agilent; Santa Clara, CA, USA), measured using a Seahorse XFe24 extracellular flux analyzer (Agilent). NCMs were plated on provided 24 well plate coated in fibronectin, plating 3–5 replicates per sample, and cultured for 3 days in 20% FBS-DMEM with 20 μM Ara-C. This time period allowed for the non cardiomyocyte cells to become metabolically inactive after the isolation procedure. On the day of measurement, cells were washed with XF assay medium (Agilent) supplemented with 25 mM glucose, 1 mM sodium pyruvate and 2 mM L-glutamine, and replenished with the same media and incubated at 37 °C without CO_2_ an hour prior to measurement. Injection scheme is represented in Fig. 1a, with concentrations at 1.5 μM oligomycin, 1 μM FCCP, 1 μM Rotenone and 1 μM antimycin A. Normalization to μg DNA/well was performed by phenol:chloroform extracting DNA from each well, and measuring DNA concentration using the Quant-iT PicoGreen dsDNA assay kit (Thermo Fisher Scientific), following the manufacturers protocol.

Rates were averaged between the 3–5 replicate plated wells per sample, giving one averaged read per sample n. Calculations for basal and maximal respiration, ATP production, spare respiratory capacity, and coupling efficiency were performed as recommended by Agilent:

Non-mitochondrial oxygen consumption (NMOC) = [min. Rate measurement after rotenone/antimycin A injection].

Basal respiration = [last rate measured before first (oligomycin) injection] - NMOC.

Maximal respiration = [max. Rate measured after FCCP injection] – NMOC.

ATP production = [last rate measured before oligomycin] – [min. Rate measurement after oligomycin injection].

Spare respiratory capacity = [maximal respiration] – [basal respiration].

Coupling efficiency = [ATP production rate] / [basal respiration] × 100.

Statistical comparisons were performed using a t-test between the measurements made in male DE vs. FA NCMs (*n* = 7, *n* = 8 respectively).

### Reduced representation bisulfite sequencing

DNA was isolated from frozen PND0 isolated cardiomyocytes using Qiagen (Germantown; MD, USA) DNeasy Blood and Tissue kit, according to the manufacturer’s protocol. For each sequencing sample, NCMs from one male neonatal heart were isolated and used for each n (*n* = 4 for FA, DE). 500 ng of DNA was digested overnight with MspI (New England BioLabs Inc., Ipswich, MA, USA) and then directly prepared with the KAPA Hyper Library prep protocol (KAPA Biosystems, Wilmington, MA, USA). Samples were then adapter-ligated using SeqCap Adapters (Roche-Nimblegen, Pleasanton, CA, USA), and cleaned using a 2.5X post-ligation Agencourt AMPure XP bead cleanup (Beckman Coulter, Indianapolis, IN). Size selection was performed to a size range of 160 bp–340 bp on a 2% Pippin Prep gel (Sage Science, Beverly, MA, USA), followed by bisulfite conversion using the Zymo EZ DNA Methylation Lightning kit (Zymo Research, Irvine, CA, USA). The converted DNA was then put back into the KAPA Hyper Library prep protocol at the library amplification step, and amplification was performed with 19 PCR cycles using 2X KAPA Hifi HotStart Uracil+ ReadyMix (KAPA Biosystems, Wilmington, MA, USA). Post-amplification cleanup was performed with 0.8X Agencourt AMPure XP beads (Beckman Coulter). Library size distributions were validated using the Agilent High Sensitivity D1000 ScreenTape run on an Agilent 2200 TapeStation (Agilent Technologies, Inc., Santa Clara, CA, USA). Additional library QC, blending of pooled indexed libraries, and cluster optimization was performed using Life Technologies- Invitrogen Qubit® 2.0 Fluorometer (Life Technologies-Invitrogen, Carlsbad, CA, USA). Sample libraries were sequenced on the Illumina HiSeq 2500 (Illumina; San Diego, CA, USA).

Sequencing reads were processed by discarding the low quality reads using Illumina’s base call quality filter, and raw reads were trimmed of adapter sequences and flanking low quality base reads using the trimmomatic read trimmer [65], discarding any reads less than 25 nt, after which there were between 13.6–31.4 million reads per sample. Methylation status for each CpG was estimated by aligning reads to the mm10 genome using the Bismark aligner [66], read into R using the Bioconductor bsseq package [67], and differential methylation was called using the Bioconductor DSS package [68]. There were between 2.85 and 4.47 million CpGs per sample. Low read coverage CpGs were filtered, based on the criteria that a CpG had to have read coverage > 5 in at least half of the samples, reducing the total number of CpGs to ~ 646 thousand. After this filtering, median read depth per CpG was 8–25. We then selected a set of differentially methylated regions, based on a set of criteria; to be considered a differentially methylated region (DMR), a) the region had to be at least 50 bp in length with at least 3 CpGs present, and b) at least 50% of the CpGs in the region had to be significant, based on a *p* < 0.001 and show a minimum 10% difference in methylation. Any significant DMRs within 100 bp of each other were merged to a single DMR. Methylation and gene expression data were from different mice, so it wasn’t possible to do any direct analysis between the two. We instead simply looked for any genes within 1 Mb of each DMR that were differentially expressed with an inverse sign (e.g., genes that increase expression as methylation decreases and vice versa).

## Abbreviations

*Acacb*: acetyl-CoA Carboxylase Beta
*Cox4i2*: cytochrome c oxidase subunit 4 isoform 2
CpG: 5′-Cytosine-phosphate-Guanine-3′
DE: diesel exhaust
DMR: differentially methylated region
DNMT: DNA methyltransferase
FA: filtered air
FCCP: carbonyl cyanide-*p*-trifluoromethoxyphenylhydrazone
*Gng11*: G protein subunit gamma 11
NCM: neonatal cardiomyocyte
PAH: polycyclic aromatic hydrocarbon
PM_10_: particulate matter with a diameter of 10 μm or less
PM_2.5_: particulate matter with a diameter of 2.5 μm or less
PND: post-natal day
RRBS: reduced representation bisulfite sequencing
TAC: transverse aortic constriction
